# The linkage between decision-making and bodily states: an investigation using an emotional startle reflex paradigm and the Iowa Gambling task

**DOI:** 10.1007/s00426-025-02114-3

**Published:** 2025-04-05

**Authors:** Azahara Miranda, Stefan Duschek, José Luis Mata

**Affiliations:** 1 Loyola University, Córdoba, Spain; 2 UMIT Tirol—University of Health Sciences and Technology, Hall in Tirol, Austria; 3 Mind, Brain, and Behavior Research Center (CIMCYC), University of Granada, Granada, Spain

## Abstract

Theories such as the somatic marker hypothesis posit that emotions and feedback from bodily states support higher cognition and decision-making. This study investigated the connection between decision-making and activity of the startle reflex, a defense reflex that is sensitive to emotional states. Decision-making was assessed using the Iowa Gambling Task (IGT), which simulates real-life decision-making with respect to complexity and uncertainty. The startle reflex was quantified, via electromyography, as the eyeblink following intense noise stimulation during the viewing of pleasant, neutral, and unpleasant emotional pictures. Forty-two healthy participants were classified according to their performance on the IGT using the median-split method. Overall, the startle amplitude was lower during pleasant and higher during unpleasant pictures than during neutral pictures. Participants with high IGT performance exhibited smaller response amplitudes than those with low IGT performance, independent of picture valence. Furthermore, inverse linear associations were seen between IGT performance and response amplitudes. The association between decision-making and startle reflex activity may be mediated by individual differences in emotional state. According to previous studies, a positive emotional state, as opposed to a negative emotional state, relates to smaller startle amplitudes and a preference for decision-making strategies based on intuition and body-related information (i.e., somatic markers), which are beneficial in situations involving complex and uncertain decisions. Moreover, an impact of individual differences in prefrontal cortex function on decision-making and startle reflex activity is feasible. The startle paradigm may be a useful tool to investigate interactions between bodily states and higher-order cognitive processing in future research.

## Introduction

As a crucial ability in daily life, decision-making refers to a set of cognitive processes enabling selection of an advantageous response from among an array of available options (Fellows, 2004; Gold & Shadlen, 2007). Psychological research suggested strong involvement of emotional experience in these processes (see Angie et al., 2011 for an overview). Positive or negative emotions, as well as emotional regulation abilities, may influence appraisal of the potential consequences of a decision and their respective likelihoods (Werner et al., 2009a). For example, anxiety modulates the tolerance for risk in experimental decision-making tasks, where highly anxious individuals tend to choose safer options than low-anxiety individuals (Maner et al., 2006; Miu et al., 2008). In further studies, positive affect was related to higher, and negative affect to lower, success levels in decision-making tasks (Buelow & Suhr, 2012; de Vries et al., 2008; Suhr & Tsanadis, 2006). The personality systems interaction theory posits that during negative affective state individuals tend to prefer analytic strategies of information processing, whereas positive emotional state relates to a more holistic processing and intuitive decision-making (Kuhl, 2000).

Current theories emphasize the role of interactions between the brain and body in emotionally guided decision-making (Damasio, 1994; Thayer et al., 2009). The somatic marker hypothesis may be most influential in this field (Bechara & Damasio, 2004). The theory posits that neural representations of physiological conditions (somatic markers) evoke feeling states that in turn support cognition, decision-making and behavioral adjustment. During decision situations, somatic markers (e.g., changes in heart activity or muscular tension) are generated together with emotional responses and connected to specific behavioral options. They are stored in memory and reactivated when similar situations occur, together with the corresponding options and likely outcomes. As such, somatic markers support decision-making via the endorsement of advantageous and rejection of disadvantageous choices. According to the theory, somatic markers are represented and regulated in central nervous emotion circuits, especially in the ventromedial prefrontal cortex (VMPFC). In decision situations, they can be evoked in two different ways. Within the “body loop”, a particular somatic state is elicited and projected to the cortex by somatosensory afferents; and in the “as-if body loop”, the representation of the somatic state is activated in somatosensory brain areas without generating an actual peripheral physiological change. These processes may occur with or without conscious awareness. Somatic markers are believed to be particularly helpful in situations characterized by high uncertainty and complexity, where they enable rapid experience-driven decision-making (Bechara & Damasio, 2004).

The Iowa Gambling Task (IGT) is a well established decision-making paradigm developed in the context of the somatic marker hypothesis (Bechara, 2007). It resembles real-life decision situations in terms of the uncertainty of outcomes and variable positive or negative consequences. The IGT requires the selection of cards from four decks (A, B, C, D), where each move can be associated with monetary gain or loss. Two decks (A, B) provide high gains and high losses. However, if these decks are selected continuously, a net loss results, such that they are disadvantageous in the long run. The two other decks (C, D) are associated with small gains and small losses but a gain results if they are chosen continuously, making them advantageous. Commonly, subjects acquire the optimal strategy to maximize total gain (i.e., choosing decks C and D and avoiding decks A and B) during execution of the task. Patients with damage to the VMPFC, where somatic markers are believed to be processed, showed impaired decision-making as they continuously chose cards from the disadvantageous decks instead of increasing the number of selections from the advantageous ones (Bechara et al., 1994, 1999, 2000). Moreover, these patients generated smaller skin conductance responses (SCRs) than healthy individuals, especially in the period preceding card selection (anticipatory SCRs), which was interpreted as suggestive of impaired development of somatic markers.

In consideration of the relevance of emotional and somatic processes to decision-making, this study investigated the implications of individual differences in the startle reflex for behavior on the IGT. This defense reflex is typically assessed as the eyeblink that occurs after sudden and intense noise stimulation (Lang et al., 1990). Its properties can be quantified via electromyography (EMG) at the orbicularis oculi muscle and according to the affective state (Bradley & Lang, 2016; Oskarsson et al., 2021). Numerous studies using emotional pictures demonstrated that the startle reflex response progressively increases during the presentation of pleasant, neutral and unpleasant pictures (e.g., Aluja et al., 2018; Bradley et al., 2001, 2006; Larson et al., 2000). The same changes can be induced using emotional stimuli like movie scenes, sounds or odors differing in pleasantness (Bradley & Lang, 2000; Ehrlichman et al., 1995; Jansen & Frijda, 1994). Due to its sensitivity to affective valence, the startle reflex is regarded as a reliable physiological marker of positive and negative emotional state (Grillon & Baas, 2003; Lang et al., 1990).

Several studies have investigated the connection between affective state and IGT performance. Building on reports of associations between negative mood and riskier judgements and behaviors (Arkes et al., 1988; Nygren, 1998), Suhr and Tsanidis (2006) demonstrated that high negative affect, quantified with the Positive and Negative Affect Schedule (PANAS, Watson et al., 1988), was related to poor IGT performance independent of personality characteristics. Similarly, Buelow and Suhr (2012) reported that individuals scoring high for negative affect on the PANAS scale made more disadvantageous and less advantageous decisions. De Vries et al. (2008) investigated the effects of naturally occurring differences in emotional state, and experimental manipulations thereof, on behavior in the IGT. Both approaches yielded evidence of better performance during a positive than negative emotional state. The association between emotional state and decision-making was explained based on the somatic marker hypothesis; it was argued that a negative affective state results in a tendency toward careful analysis of available information, whereas during a positive affective state individuals tend to rely on intuition and “gut feelings” (Bolte et al., 2003; de Vries et al., 2008; Wagar & Dixon, 2006).

It is important to note that the described findings of connections between affective state and IGT performance were exclusively based on questionnaire ratings for affect (Buelow & Suhr, 2012; de Vries et al., 2008; Suhr & Tsanidis, 2006). In contrast, psychophysiological parameters of positive and negative affect have not yet been used in this context. This must be regarded as a limitation of the research field, as, by definition, affect constitutes a multidimensional phenomenon involving physiological components in addition to subjective experience (Bradley & Lang, 2016; Niedenthal et al., 2025). The present study aimed to fill this gap in the literature by using the startle response as a psychophysiological indicator of affect and relating its strength to decision-making performance on the IGT. The reliable variation in the startle response according to affective valence makes it particularly suitable for this purpose. While other psychophysiological parameters like skin conductance, heart rate or muscle tension merely reflect emotional arousal, the startle reflex is unique in indexing the valence dimension of emotion (Grillon & Baas, 2003; Kuhn et al., 2000).

The startle paradigm applied in the study involved eyeblink responses to noise stimuli during the viewing of pleasant, neutral and unpleasant affective pictures. The main hypothesis pertained to a connection between startle reflex amplitude and decision-making performance assessed via the IGT. Precisely, individuals exhibiting higher task performance (i.e., more advantageous and less disadvantageous decisions) were expected to be characterized by smaller reflex responses than those with low decision-making performance (i.e., by more disadvantageous and less advantageous decisions). This hypothesis is informed by the described dependence of startle amplitude on affective state; while larger amplitudes are regarded as a psychophysiological correlate of negative affective state, positive affective state is accompanied by smaller amplitudes (Bradley & Lang, 2016; Grillon & Baas, 2003). On the other hand, negative affective state is associated with more risky decisions (Nygren, 1998); and individuals performed better on the IGT during positive than during negative affective state (Buelow & Suhr, 2012; de Vries et al., 2008). Altogether, these observations give rise to the prediction that larger startle responses relate to poorer IGT performance than smaller startle responses.

## Methods

### Participants

In total, 42 students from the Bachelor of Psychology program of the University of Granada (Spain) participated in the study. All of them were native speakers of Spanish. They were assigned to two groups according to their performance on the IGT (high IGT performance vs. low IGT performance). Therefore, the group was split based on the median IGT score (n = 21 per group; see the Iowa Gambling Task section for computation of the IGT score). The group demographics were as follows: high performance group, 14 men, 7 women; age, M = 20.57 years, SD = 3.30 years; IGT score, M = 29.90, SD = 25.40; low performance group, 16 men, 5 women; age, M = 20.24 years, SD = 4.26 years; IGT score, M = − 19.43, SD = 17.86. Participants were only included if they did not suffer from any relevant physical or mental disorders and did not use any psychoactive drugs.

Sample size estimation was based on previous studies concerning associations between affect related variables and IGT performance, which revealed-medium-to-large effect sizes overall (e.g., Buelow & Suhr, 2012; De Vries et al., 2008; Suhr & Tsanidis, 2006). Assuming an effect size (*η*_*p*_^*2*^) of 0.30, an alpha error of 5% and a beta error of 30% for a mixed ANOVA (two independent groups, three repeated measurements), power analysis with G*Power (ver. 3.1.9.7) (Faul et al., 2007) revealed a required sample size of 40 participants. For the same ANOVA model, sensitivity analysis based on an alpha error of 5%, a beta error of 30% and 40 participants suggested that effects ≥ 0.29 (*η*_*p*_^*2*^) would be detected. Regarding correlation analysis, in a sample of 40 participants (alpha = 5%, beta = 30%), correlation coefficients ≥ 0.26 would reach significance (one-tailed testing).

### Iowa Gambling task

**Fig. 1:**
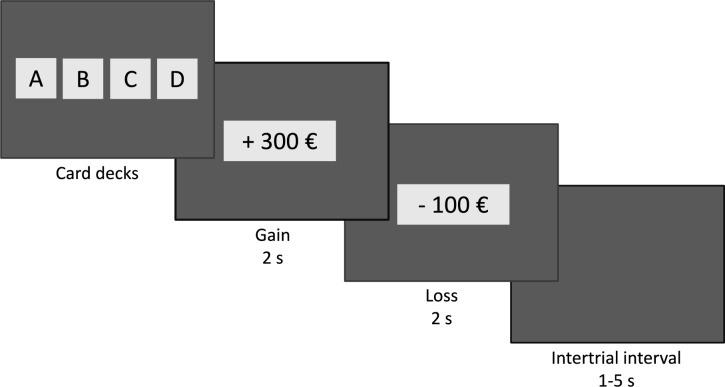
Task scheme of the IGT

The standard version of the IGT described by Bechara (2007) was used in the study (see Fig. 1 for task scheme). It was presented on a 22-inch monitor using E-Prime 2.0 software (Pinker, 2015). At the beginning of each trial, four virtual card decks (decks A, B, C, D) were concurrently displayed on the screen. Participants selected a card from one deck using the left mouse button. After the selection, the decks disappeared, and two numbers were sequentially displayed for 2 s each. The first number provided information about monetary gain (e.g., + 100 €), and the second one information about loss (e.g., − 300 €), in the respective trial. Each trial was associated with either overall gain (e.g., gain + 300 €, loss–200 €) or overall loss (e.g., gain + 100 €, loss–200 €). In total, 100 trials were performed; the task was presented in blocks of 20 trials each with short breaks between them. The intertrial intervals ranged between 1 and 5 s. Decks A and B provided large gains but also large losses. If chosen continuously, these decks led to a net loss (disadvantageous decks). Decks C and D provided modest gains and modest losses, and the choice thereof resulted in long-term net profit (advantageous decks). The current monetary balance was continuously shown on the screen. Participants started the task with a 2000 € credit of play money. All instructions were given in oral and written form and corresponded to those described by Bechara (2007). In short, participants were informed that in each trial they had to choose a card from one of four decks, that each choice was associated with monetary gain and loss and that the goal of the task was to maximize overall gain. Individual task performance was indexed by the IGT score, computed according to the formula (C + D)–(A + B) (Bechara, 2007). The computation of the score was based on all 100 task trials. Preliminary data analysis performed separately for each of the five blocks did not reveal differential connections with the startle reflex; therefore, the final data analysis was only conducted for the 100 trials in their entirety (see Results section for learning curves across the five blocks).

### Startle reflex paradigm

**Fig. 2:**
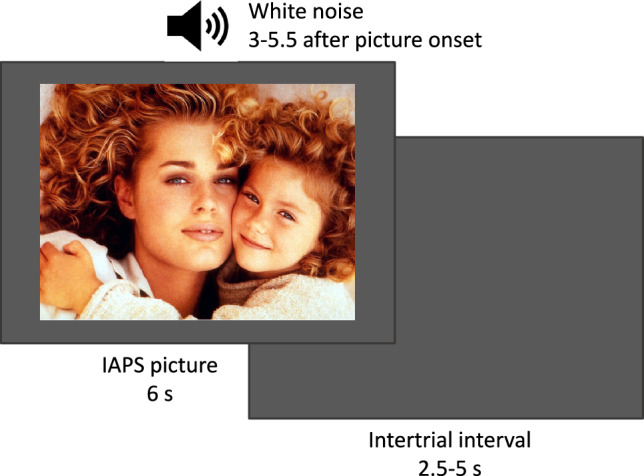
Stimuli and timing of the startle paradigm

The startle paradigm was also controlled using E-Prime 2.0 software (Pinker, 2015); stimulus markers were recorded by a Biopac MP 100 system (Biopac Systems Inc., Goleta, CA). The startle response was triggered by 105 dB white noise stimuli of 50 ms duration, recorded in a wav file, amplified with an IMG Stage Line 2000 amplifier (Monacor, Bremen, Germany) and presented through AKG K240 headphones (Harman, Inc., Garching, Germany; see Fig. 2 for stimuli and timing). A total of 27 noise stimuli were delivered while participants were viewing affective pictures.

Therefore, 45 pictures were selected from the International Affective Picture System (IAPS; Moltó et al., 1999). Each picture (size 640 × 480 pixels) was displayed for 6 s on a 22-inch monitor at a viewing distance of 50 cm. They depicted persons either in pleasant (e.g., erotica), neutral (e.g., formal interactions) or unpleasant (e.g., mutilations) situations. Per affective category, 15 pictures were chosen with the following codes: pleasant, 4607, 4608, 4651, 4652, 4658, 4659, 4664, 4670, 4680, 4687, 4800, 4810, 4687, 4800, 4810; neutral, 2190, 2214, 2230, 2372, 2383, 2440, 2480, 2485, 2495, 2514, 2840, 7550, 2514, 2840, 7550; unpleasant, 3000, 3010, 3030, 3051, 3053. 3071, 3100, 3110, 3150, 3168, 3170, 3400, 3168, 3170, 3400. The mean valence and arousal ratings taken from the normative data were as follows (Moltó et al., 1999); pleasant: valence = 7.08, arousal = 5.73; neutral: valence = 5.28, arousal = 2.84; unpleasant: valence = 1.91, arousal = 6.03. Pictures from the three categories were equivalent in brightness and contrast. During the presentation of 9 of the 15 pictures in each category (random assignment), noise stimuli were delivered 3 s, 3.5 s, 4 s, 4.5 s, 5 s or 5.5 s after picture onset. Intertrial intervals ranged between 2.5 and 5 s. Pictures were presented in blocks according to their affective valence (pleasant, neutral, unpleasant). The presentation order of the three blocks was counterbalanced across participants. Participants were asked to continuously look at the pictures.

### EMG recording

The EMG was recorded from two miniature Ag/AgCl electrodes filled with standard electrode gel and attached to the skin overlying the orbicularis oculi muscle of the left eye. One electrode was attached underneath the pupil and the second one 1 cm lateral to the outer cantus of the eye (Blumenthal et al., 2005; Tassinary et al., 2016). The signal was amplified using an EMG100C amplifier (Biopac MP 100) with a gain of 500 and a band pass filter with a low cut-off of 10 Hz and a high cut-off of 500 Hz. Data were stored at a sampling rate of 1000 Hz using AcqKnowledge 3.9 software (Biopac Systems Inc.).

The amplitude of the startle response to each of the 27 noise stimuli was quantified as the absolute difference (in µV) between the mean value of a 20 ms baseline starting at stimulus onset and the maximal value during the period from 20 to 150 ms after stimulus onset (Blumenthal et al., 2005; Tassinary et al., 2016). The amplitudes were averaged across the nine trials of each affective category. No data were removed due to artefacts in the EMG.

### Procedure

After completing the informed consent form, participants performed the IGT in the form described above. Subsequently, the EMG electrodes were attached, and the startle paradigm was executed. Finally, participants evaluated the IAPS pictures on the valence and arousal dimensions of the Self-Assessment Manikin scale (Moltó et al., 1999). The study procedure complied with the APA ethical standards and the Declaration of Helsinki. All participants provided written informed consent. The Ethical Committee of the University of Granada approved the study protocol (IRB#2994/CEIH/2022).

### Data analysis

For statistical analysis of the startle reflex amplitude, a mixed ANOVA was computed with the between-subjects factor of Group (high IGT performance, low IGT performance) and the within-subjects factor of Picture Category (pleasant, neutral, unpleasant). Due to the methodological restrictions associated with group formation according to median split of the IGT score, an addition model was computed with the within-subjects factor of Picture Category and the (continuous) IGT score as covariate. ANOVAs with the factors Group and Picture Category were performed for the SAM ratings of valence and arousal. Only ratings on the pictures during which noises were delivered were included in this analysis. In the ANOVAs, Greenhouse–Geisser correction was applied where necessary. Post-hoc analyses were performed using repeated-measures F-tests. Partial η^2^ was used as the measure of effect size. To evaluate linear associations between IGT performance and startle reflex amplitude separately for the three picture categories, parametric correlations were computed and tested for significance (one-tailed testing). Alpha was set at 0.05 in all analyses. Statistical analyses were carried out using IBM SPSS Statistics (ver. 24; IBM Corp., Armonk, NY).

## Results

### IGT learning curves

**Fig. 3:**
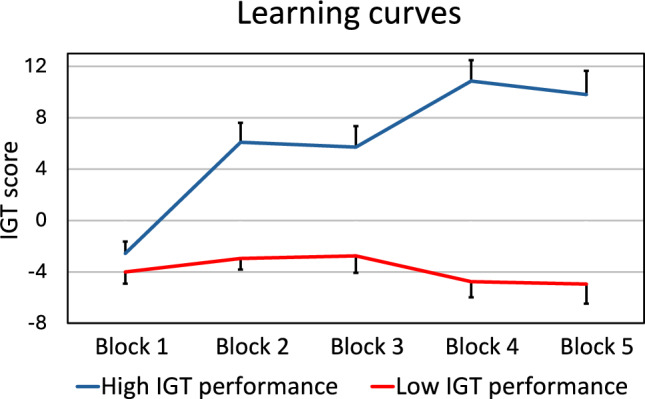
Learning curves on the IGT for participants with high and low task performance (bars represent standard errors of the mean)

Figure 3 displays the learning curves for the IGT of the two study groups classified according to their task performance. The IGT score is displayed for the five blocks of the task, each comprising 20 trials (computed according to the formula (C + D) – (A + B)). While the score showed a strong increasing trend across the five blocks in the group with high performance, it remained virtually unchanged in the group with low performance.

### Startle reflex amplitude

**Fig. 4:**
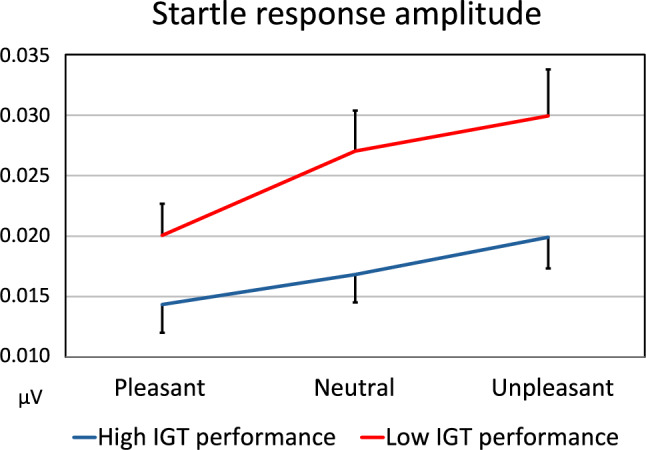
Startle reflex amplitude for the three affective picture categories in participants with high and low IGT performance (bars represent standard errors of the mean)

Figure 4 displays the startle reflex amplitude for both groups and the three affective picture categories. The Group × Picture Category ANOVA revealed a main effect of the Group factor (*F*(1, 40) = 4.91,* p* = 0.032, η_p_^2^ = 0.11); the amplitude was larger overall in the group with low IGT performance than in the group with high IGT performance. A main effect of Picture Category indicated that the amplitude differed according to the affective valence of the pictures in the entire sample (*F*(2, 80) = 26.92, *p* < 0.001, *η*_*p*_^*2*^ = 0.40). It was larger for unpleasant than pleasant (*F*(1, 41) = 46.28, *p* < 0.001, *η*_*p*_^*2*^ = 0.53) and neutral (*F*(1, 41) = 7.94, *p* = 0.007, *η*_*p*_^*2*^ = 0.16) pictures, and for neutral than pleasant (*F*(1, 41) = 20.05, *p* < 0.001, *η*_*p*_^*2*^ = 0.33) pictures. The Group × Picture Category interaction did not reach significance (*F* (2, 80) = 2.87, *p* = 0.062, *η*_*p*_^*2*^ = 0.067). The ANCOVA revealed a significant effect of the covariate IGT score (F[1, 40] = 6.41, p = 0.015, η_p_^2^ = 0.14), in addition to a Picture Category effect (F[2, 80] = 28.41, p < 0.001, η_p_^2^ = 0.41). The interaction effect was not significant (F[2, 80] = 2.56, p = 0.083, η_p_^2^ = 0.06). In the entire sample, the IGT score correlated negatively with the startle reflex amplitude recorded during the presentation of unpleasant (r = − 0.37, p = 0.008), neutral (r = − 0.39, p = 0.006) and pleasant (r = − 0.31, p = 0.025) pictures.

### Self-assessment manikin (SAM) scale

Table 1 includes the SAM ratings for the affective pictures (higher values denote more positive valence and greater arousal). The ANOVA for the valence ratings yielded main effects of Group (*F*(1, 40) = 7.86,* p* = 0.008, η_p_^2^ = 0.16) and Picture Category (*F*(2, 80) = 266.59, *p* < 0.001, *η*_*p*_^*2*^ = 0.87); the interaction effect was not significant (*F*(1, 40) = 2.65,* p* = 0.085, η_p_^2^ = 0.062). Valence ratings were most positive for pleasant pictures, followed by neutral and unpleasant pictures (p < 0.001 for all differences). The Group effect denotes more positive picture evaluations in participants with high IGT performance. The ANOVA for the arousal ratings revealed a main effect of Picture Category (*F*(2, 80) = 50.14, *p* < 0.001, *η*_*p*_^*2*^ = 0.56). The Group (*F*(1, 40) = 0.04,* p* = 0.84, η_p_^2^ = 0.001) and interaction (*F*(1, 40) = 2.87,* p* = 0.062, η_p_^2^ = 0.067) effects were not significant. Pleasant and unpleasant pictures were rated as more arousing than neutral pictures (pleasant vs. neutral: *F*(1, 41) = 86.90, *p* < 0.001, *η*_*p*_^*2*^ = 0.68; unpleasant vs. neutral: *F*(1, 41) = 75.70, *p* < 0.001, *η*_*p*_^*2*^ = 0.65); arousal ratings did not differ between pleasant and unpleasant pictures (*F*(1, 41) = 0.56, *p* = 0.46, *η*_*p*_^*2*^ = 0.013).

**Table 1:** Mean (SD in parentheses) Self-Assessment Manikin (SAM) ratings of valence and arousal in both study groups; higher values denote more positive valence and greater arousal. The presented data are based only on the pictures during which noises were delivered

	Pleasant	Neutral	Unpleasant
SAM valence			
High IGT performance	7.80 (0.96)	5.26 (0.60)	1.96 (1.21)
Low IGT performance	6.79 (1.48)	5.35 (0.88)	1.63 (0.76)
SAM arousal			
High IGT performance	5.67 (2.48)	2.42 (1.28)	6.48 (2.10)
Low IGT performance	5.87 (1.93)	3.29 (1.38)	5.66 (2.24)

## Discussion

This study investigated for the first time the possible implication of the activity of the startle reflex for decision-making. The eyeblink response to aversive noise stimulation was recorded during the viewing of pleasant, neutral and unpleasant affective pictures in healthy individuals classified according to their performance on the IGT. Participants with low IGT performance exhibited a larger startle response overall than those with high IGT performance. In the entire sample, the response amplitude increased from pleasant to unpleasant pictures. Furthermore, inverse linear associations were seen between IGT performance and the response amplitude during the viewing of pleasant, neutral and unpleasant pictures.

The startle reflex involves an automatic response to sudden and intense stimulation that is associated with the mobilization of defensive systems and unpleasant subjective experience (Lang et al., 1990). It is regarded as a protective mechanism, facilitating coping with potential threats by interrupting current behaviors and focusing attention on the source of the threat (Oskarsson et al., 2021). Though the startle reflex affects the entire body, the eyeblink constitutes the fastest and most reliable component thereof and is therefore commonly assessed in psychophysiological research (Bradley & Lang, 2016). The affective modulation of the startle reflex amplitude observed in the present study is in accordance with the literature. A large database substantiates startle potentiation during negative affective states; despite its somewhat smaller extent, attrition of the reflex during positive affect is also widely acknowledged (see Bradley & Lang, 2016 and Grillon & Baas, 2003 for overview). According to the SAM ratings, pleasant and unpleasant pictures were perceived as more arousing than neutral pictures. Nevertheless, the startle response progressively increased across pleasant, neutral and unpleasant pictures. This supports the notion that the reflex changes as a function of emotional valence and is virtually unaffected by arousal (Bradley & Lang, 2016; Grillon & Baas, 2003). According to the motivational priming hypothesis, defensive mechanisms, such as the startle reflex, are automatically primed during aversive experience; by contrast, positive emotional experience is connected to the inhibition of defensive mechanisms and activation of appetitive motivation (Lang, 1995; Lang et al., 1990).

As illustrated by the learning curves, the group with higher IGT scores showed a strong increase in advantageous, and decrease in disadvantageous, decisions across the five blocks of the task. In contrast, no learning was evident in the group with lower IGT scores. The knowledge that differences in the startle amplitudes constitute a psychophysiological correlate of positive and negative affective states may be relevant to the association of the startle reflex with decision-making. As initially stated, individuals performed poorer on the IGT during negative than positive affective state (Buelow & Suhr, 2012; de Vries et al., 2008). In addition, there is evidence that positive and negative emotional states are associated with different cognitive processing modes and specific behaviors in decision situations (Angie et al., 2011; Bolte et al., 2003). According to the personality systems interaction theory, a negative emotional state supports an analytic processing mode, whereas positive emotional state relates to a holistic processing mode and more intuitive decision-making (Kuhl, 2000). In accordance with this reasoning, Bolte et al. (2003) reported that positive affect improved participants´ ability to make intuitive judgments about the semantic coherence of verbal stimuli; negative affect had the opposite effect. This may be relevant to the dependence of IGT performance on emotional states. With reference to the somatic marker hypothesis, it was claimed that under a positive emotional state, decisions are more likely to be made based on feelings and information arising from within the body (Bolte et al., 2003; de Vries et al., 2008; Wagar & Dixon, 2006). In complex and uncertain situations, such as that simulated by the IGT, unambiguous information enabling deduction of a rational strategy is unavailable; thus, affectively guided and intuitive strategies are more effective (Wagar & Dixon, 2006). This is illustrated by the observation that healthy individuals tend to decide advantageously on the task before being aware of the advantageous strategy (Bechara et al., 1997). Furthermore, somatic markers (represented by anticipatory SCRs) are developed at a relatively early stage of task execution, and their generation relates positively to performance (Bechara et al., 1996; Wagar & Dixon, 2006). In contrast, explicit conscious knowledge of the relative value of the four card desks is gained in later stages (Bechara et al., 1997; Maia & McClelland, 2004). Considering this, in the IGT, decisions driven by emotion and “gut feelings” (i.e., somatic markers), such as those related to a positive emotional state, may be superior to decisions based on analytic strategies, such as those related to a negative emotional state.

As a defense mechanism, the startle reflex is closely associated with the processing of threat and anxiety; hence, individual differences in its magnitude reflect differences in threat-related physiological reactivity (Lang et al., 1990). It may be that individuals characterized by high threat-related reactivity also exhibit stronger bodily responses during the IGT, especially when considering risky decisions (decks A and B). In terms of the somatic marker hypothesis, highly reactive individuals would command a larger amount of somatic information, helping to avoid risky and thus disadvantageous decisions. Evidently, this was not the case in this study, as greater startle responses were associated with more frequent instead of less frequent disadvantageous decisions. It may be hypothesized that strong physiological activation does not necessarily imply improved emotional guidance in decision-making. To serve as somatic markers, physiological changes must be linked to behavioral options and, in addition, must be accessible to central nervous and mental processing. This is illustrated by a study concerning the role of interoceptive sensibility in decision-making (Werner et al., 2009b). Performance on the IGT was compared between individuals with high and low interoceptive sensitivity, assessed via a heartbeat perception task (Schandry, 1981). While high interoceptive sensitivity was associated with better IGT performance, heart rate recorded during anticipation of decisions and feedback pertaining to gain and loss did not differ between individuals with high and low interoceptive sensitivity. This underlines that decision-making varies according to the accessibility of somatic feedback rather than its actual magnitude.

The extent of affective modulation of the startle reflex was unrelated to IGT performance in this study. Alterations in affective modulation were mainly observed in clinical conditions. While individuals with specific phobias exhibited greater affective modulation than controls (Lang et al., 2005), the opposite was reported, for example, in depression and sociopathy (Kaviani et al., 2004; Oskarsson et al., 2021). This was discussed in terms of emotional and motivational dysregulations in the pathogenetic mechanisms (Grillon & Baas, 2003). Some studies also related individual differences in affective startle modulation to psychological features in healthy individuals (Cook et al., 1991; Grüsser et al., 2006; Vaidyanathan et al., 2008); however, according to the present study, individual differences in the response magnitude seem more relevant to decision-making than variations therein according to emotional stimuli.

In addition to the impact of emotion on the use of emotional and somatic information during decision-making, top-down effects of high-order cognition on emotional processing should be taken into account in the connection between the startle reflex and decision-making. Decision-making involves weighing multiple alternatives and selecting an advantageous option while reflecting on potential positive and negative consequences; these abilities strongly depend on cognitive control (Fellows, 2004; Miller & Cohen, 2001). As a neural correlate of cognitive control, the prefrontal cortex plays a key role in decision-making (Miller & Cohen, 2001; Miyake et al., 2000). The neurovisceral integration model posits a close interaction between the prefrontal cortex and activity in limbic structures, especially the amygdala (Thayer & Lane, 2008). In turn, the amygdala is relevant to the startle response. Though the startle reflex is characterized as a brainstem reflex, neuroimaging studies in humans suggest an important role of the amygdala in its affective modulation (Anders et al., 2004). Kuhn et al. (2020) conducted brainstem- and amygdala-specific fMRT during a paradigm in which the startle reflex was elicited during affective picture presentation. They identified a neural pathway including the nucleus reticularis pontis caudalis (PnC) in the brainstem and the centromedial fraction of the amygdala (CMA) that underlies affective startle modulation. While the PnC is crucial to the elicitation of the eyeblink response, affect-related activity in the CMA seems to determine the magnitude of PnC activation. Moreover, it is well-known that the amygdala becomes active during conditions of uncertainty; in terms of negative bias, it preferentially responds to threatening information (Cunningham et al., 2008). Amygdala activation is regarded as part of a “default response” to uncertainty that corresponds to defense mechanisms like the startle reflex or the fight-and-flight response, protecting the organism and mobilizing energetic resources to ensure survival. Importantly, amygdala activity is inhibited most of the time via projections from the prefrontal cortex. However, inhibition strongly varies among individuals. Differences in prefrontal cortex function modulate top-down control of the amygdala, where poorer prefrontal function is accompanied by weaker inhibition and thus greater amygdala activity (Thayer & Lane, 2008). Considering this, larger startle response magnitudes may relate to amygdala disinhibition and greater threat processing due to poorer prefrontal function.

According to the SAM scale of valence, the participants in this study with high IGT performance perceived the emotional pictures as more pleasant than those with low IGT performance. This suggests a tendency toward more positive emotional reactivity in these individuals, which does not conflict with the proposed interpretations. A relevant limitation of this study pertains to the lack of assessment of variables that would have facilitated interpretation of the results in terms of relevant psychophysiological mechanisms. Measurements of emotional state during the IGT and subjective responses to noise stimuli could have substantiated the hypothesized role of individual differences in emotional state in the detected associations. In addition, recording of a parameter of autonomic function like electrodermal activity or heart rate during the IGT would have been beneficial. In future research, the role of prefrontal cortex function in the linkage between decision-making and startle reflex activity could be investigated by recording vagally mediated heart rate variability (vmHRV). vmHRV constitutes an index of the integrity of prefrontal processing (Berntson et al., 2016; Thayer & Lane, 2008). Moreover, individual differences in vmHRV are associated with cognitive control and emotional regulation (Appelhans & Luecken, 2006; Bair et al., 2020, 2022; Hansen et al., 2003). Another restriction pertains to the comparison of the groups composed according to median split. It has been shown that this method may lead to increased effect sizes for psychophysiological variables (Morriss et al., 2024). However, our conclusions are also supported by the findings from the ANCOVA and correlation analysis, in which the IGT score was treated as a continuous variable.

In sum, this study revealed evidence of a connection between startle reflex activity and decision-making in healthy individuals. This association may be explained by individual differences in emotional state, which affect the startle response and modulate cognitive processing modes and decision-making strategies. Moreover, top-down effects of prefrontal cortex function on decision-making, emotional processing and the startle reflex are feasible. The study adds to the research on the peculiarities of the startle reflex in mental disorders like post-traumatic stress disorder, obsessive compulsive disorder, psychopathy or substance abuse (Jurado-Barba et al., 2017; Kumari et al., 2001; Medina et al., 2001; Oskarsson et al., 2021), and its associations with personality characteristics like trait anxiety, aggressiveness and reward sensitivity (Aluja et al., 2014; Blanch et al., 2014; Sege et al., 2018; Vaidyanathan et al., 2008). The findings illustrate that the startle paradigm may be a useful tool to investigate the interaction between bodily states and higher-order cognitive processing in future psychophysiological research.

## Data Availability

The research data is available on request.
